# The combined effect of determinants on coverage of intermittent preventive treatment of malaria during pregnancy in the Kilombero Valley, Tanzania

**DOI:** 10.1186/1475-2875-10-140

**Published:** 2011-05-21

**Authors:** Karin Gross, Sandra Alba, Joanna Schellenberg, Flora Kessy, Iddy Mayumana, Brigit Obrist

**Affiliations:** 1 Swiss Tropical and Public Health Institute, Basel, Switzerland; 2 University of Basel, Basel, Switzerland; 3 Ifakara Health Institute, Dar es Salaam, Tanzania; 4 London School of Hygiene and Tropical Medicine, London, UK; 5 University of Basel, Institute of Anthropology, Basel, Switzerland

## Abstract

**Background:**

Intermittent preventive treatment during pregnancy (IPTp) at routine antenatal care (ANC) clinics is an important and efficacious intervention to reduce adverse health outcomes of malaria infections during pregnancy. However, coverage for the recommended two IPTp doses is still far below the 80% target in Tanzania. This paper investigates the combined impact of pregnant women's timing of ANC attendance, health workers' IPTp delivery and different delivery schedules of national IPTp guidelines on IPTp coverage.

**Methods:**

Data on pregnant women's ANC attendance and health workers' IPTp delivery were collected from ANC card records during structured exit interviews with ANC attendees and through semi-structured interviews with health workers in south-eastern Tanzania. Women's timing of ANC visits and health worker's timing of IPTp delivery were analyzed in relation to the different national IPTp schedules and the outcome on IPTp coverage was modelled.

**Results:**

Among all women eligible for IPTp, 79% received a first dose of IPTp and 27% were given a second dose. Although pregnant women initiated ANC attendance late, their timing was in line with the national guidelines recommending IPTp delivery between 20-24 weeks and 28-32 weeks of gestation. Only 15% of the women delayed to the extent of being too late to be eligible for a first dose of IPTp. Less than 1% of women started ANC attendance after 32 weeks of gestation. During the second IPTp delivery period health workers delivered IPTp to significantly less women than during the first one (55% vs. 73%) contributing to low second dose coverage. Simplified IPTp guidelines for front-line health workers as recommended by WHO could lead to a 20 percentage point increase in IPTp coverage.

**Conclusions:**

This study suggests that facility and policy factors are greater barriers to IPTp coverage than women's timing of ANC attendance. To maximize the benefit of the IPTp intervention, revision of existing guidelines is needed. Training on *simplified *IPTp messages should be consolidated as part of the extended antenatal care training to change health workers' delivery practices and increase IPTp coverage. Pregnant women's knowledge about IPTp and the risks of malaria during pregnancy should be enhanced as well as their ability and power to demand IPTp and other ANC services.

## Background

Malaria is still a major cause of morbidity and mortality in Tanzania, especially for small children and pregnant women [1]. About 1.7 million pregnant women contract malaria each year in Tanzania [2] leading to a high risk of suffering from severe anaemia, spontaneous abortion, preterm delivery, congenital infection, still birth and low-birth weight [3,4]. Malaria during pregnancy is a contributing factor to both maternal death [5] and infant morbidity and mortality [6,7]. Infection rates have been found to be especially high in women in their first and second pregnancy [4,8], but also depend on other factors such as endemicity, immunity, age, trimester and co-morbidities [9].

Committing themselves to the Abuja targets, Tanzania and other countries in sub-Saharan Africa adopted the World Health Organization's (WHO) recommendation for malaria prevention and control during pregnancy [10,11]. In areas of stable malaria transmission in sub-Saharan Africa, this means the implementation of an intervention package into the antenatal care (ANC) services including the use of insecticide-treated nets (ITN) and intermittent preventive treatment in pregnancy (IPTp) as well as effective case management of malaria and anaemia [12]. Thus, every Tanzanian pregnant woman attending an ANC clinic is entitled to receive a national voucher for a subsidized ITN, known as *Hati Punguzo *("discount card" in Swahili) and IPTp [13]. In 2003, WHO defined IPTp as the delivery of two doses of an anti-malarial to pregnant women at the beginning of the second and third trimester irrespective of the presence of signs for a malaria infection [14]. Sulphadoxine-pyrimethamine (SP) is the drug currently used for IPTp in areas of Africa where malaria is transmitted by *Plasmodium falciparum *as it has been shown to be a cheap, safe and effective single-dose treatment [12,15,16]. There is also evidence that the intervention is well accepted by women in various African settings [17,18]. Taking health workers' difficulties to assess gestational age into account, WHO modified and simplified the IPTp recommendations in 2004: "beginning of the second trimester" was replaced by "after quickening" (first noted movements of the foetus) and "third trimester" by "at least one month apart". This change in wording also implies that more than two doses can be administered [12,19]. In the context of the HIV epidemic and increasing SP resistance, discussions have arisen on the optimal number of IPTp doses required to maintain protection for the mother and her child. Based on its relatively low HIV prevalence rate of 6% [20], Tanzania is the only East-African country to keep a two dose regimen regardless of HIV status [21] as WHO recommends the introduction of a three dose regimen where HIV prevalence is above 10% [12].

**Figure 1:**
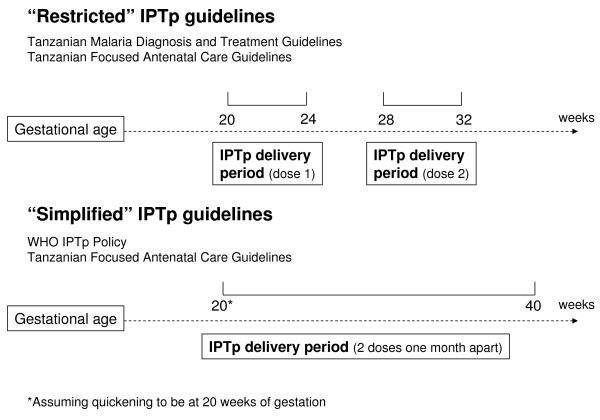
The two national IPTp guidelines

In Tanzania, two different IPTp recommendations are available and they partially disagree with current WHO recommendations (see Figure 1). Firstly, the revised national malaria diagnosis and treatment guidelines from 2005 recommend the administration of a first dose of IPTp between 20-24 weeks and a second dose between 28-32 weeks of gestation [22]. This recommendation is critically different from the current WHO recommendation that all pregnant women in areas of stable malaria transmission should receive at least two doses of IPTp after quickening and at least one month apart [12]. Secondly, based on WHO's new ANC model and drawing on the experiences from Kenya, Tanzania developed in 2002 the Focused Antenatal Care (FANC) guidelines with specific reference to malaria in pregnancy [23]. The FANC guidelines in principle follow the revised WHO IPTp recommendation by stating that IPTp "can be given at any point in pregnancy after 16 weeks as long as [the doses are] one month apart" [23: 81] and "is safe from quickening up to 40 weeks of gestation" [23: 86]. Confusingly, the guidelines also still recommend IPTp to be delivered between 20-24 weeks and again between 28-32 weeks of gestation [23: 104].

Tanzania was among the first countries to adopt IPTp as a national strategy in 2000 along with Malawi, Kenya, Uganda and Zambia [24]. ANC attendance is high with 62% of women attending at least four times [1]. Yet coverage is still far from reaching the target of 80% IPTp coverage by 2010 as proposed by the Roll Back Malaria Partnership and set in Tanzania for 2012 [11,25]. Coverage levels of the second IPTp dose are particularly low. According to the Tanzanian Malaria and HIV Indicator Survey 2007/08 57% of all pregnant women reported receiving one dose of IPTp but only 30% said they were given a second dose during an ANC visit [20]. Disappointing second dose coverage results of below 40% have also been found by other studies from Tanzania and East Africa [26-30].

Recent studies have stressed the need to investigate the impact of available guidelines on implementation, health worker practices and drug stock-outs at the health facilities in order to understand the reasons for low IPTp coverage [26-28,30,31]. However, only a few studies have addressed these factors so far. Instead, studies have associated low IPTp uptake with women's late initiation and irregular attendance of ANC services [29,32,33]. Others have investigated the relationship between IPTp uptake and women's knowledge about malaria [28,32-35]. Yet others have explored the influence of pregnant women's age, marital status, educational level, socio-economic status or parity on IPTp uptake [26,35,36]. Overall, studies with a focus on women's individual characteristics were not able to explain low IPTp levels [27-29,35,36]. Two recent studies from Tanzania demonstrated that delivery of IPTp was influenced by facility and policy level factors. Marchant et al. [30] analysed national household and facility survey data from Tanzania and identified insufficient SP stocks at the health facilities and restrictive guidelines as the main reasons for low IPTp delivery. Similarly, Anders et al. [26] argued that revised guidelines and improved drug stocks would allow for delivery of IPTp at an earlier gestational age and to increase IPTp coverage.

The aim of this paper is to assess the combined impact of women's timing of ANC attendance, health workers' IPTp delivery and different delivery schedules of national IPTp guidelines on IPTp coverage. The study used ANC card records collected during exit interviews with ANC attendees to analyze pregnant women's timing of ANC visits and health workers' delivery and timing of IPTp in relation to the main IPTp guidelines in use in Tanzania and to model the anticipated outcome of a second set of guidelines on IPTp coverage.

## Methods

### Study area

This study on IPTp coverage was conducted in the frame of a wider research project that explored determinants of access to ANC and IPTp services in the Kilombero and Ulanga Districts of the Morogoro Region in south-eastern Tanzania between April 2007 and May 2009. The two districts are divided by the floodplain of the Kilombero River which is delimited by the Udzungwa Mountains to the north and by the Mahenge Mountains to the south. Large parts of the valley are regularly flooded during the rainy season from November to May.

The study area coincides with a Demographic Surveillance System (DSS) that encompasses a total of 25 villages of the two districts with an estimated total population of 92'000 in 2008 [37,38]. In the early 1990s, malaria transmission in this area was amongst highest in Tanzania with a mean entomological inoculation rate (EIR) of over 300 infective bites per person per year [39]. Although malaria transmission in the area has been reduced substantially through the use of untreated and insecticide-treated nets and effective malaria drugs, it remains high and perennial [40,41]. Malaria is still the most commonly diagnosed cause of illness in health facilities [42], but there is recent evidence that malaria is over-diagnosed, especially in the urban and peri-urban areas (personal communication V. D'Acremont). The area is predominantly rural and households rely heavily on agriculture of rice, maize, banana and cassava. During the rice planting and harvesting seasons many people move for several months to temporary shelters in distant farming sites [43].

The Tanzanian public health system consists of a wide network of dispensaries, health centres and hospitals with each facility serving between 3,300 and 7,000 people [44]. At the time of study a total of 13 first and second-level health facilities offered regular out-patient services within the DSS area. Out of these, 12 (ten government and two faith based) facilities provided ANC services for pregnant women on a weekly or daily basis from Monday to Friday. Two district hospitals provide referral care for complicated cases. In the course of the Tanzanian Health Sector Reform the districts have introduced a cost-sharing scheme in public facilities, including an exemption policy for pregnant women and children under five years of age.

DSS records suggest that in 2008, 3,033 women became pregnant in the Kilombero and Ulanga DSS. Women reported an average of 3.1 visits to ANC clinics over the course of a pregnancy (personal communication: M. Alexander). Data collected from the Health Management Information System (HMIS) on the DSS area indicated that IPTp coverage had increased from 24% to 45% between 2006 and 2008 for one IPTp dose and from 7% to 21% for two doses (Gross, unpublished data).

### Study design and study population

The study draws on data from two study components of the larger research project: 1) in-depth interviews with health workers at the ANC clinics were conducted in June 2007 and 2) exit interviews with ANC attendees were performed over a five month period between June and October 2008.

In-depth interviews with 18 health workers at 12 ANC clinics were conducted, including all health workers routinely working at the ANC clinic and available on the day of visit. Between one to three health workers were interviewed per health facility.

A total of 440 pregnant women visiting an ANC clinic participated in the exit interviews. Ten facilities (nine government facilities and one faith based facility, five in the Kilombero district and five in the Ulanga district) were selected and visited once per month for one day. Since accessibility of health services in this rural context is constrained through seasonal conditions such as weather, agricultural work or availability of money, the exit interviews were scattered over several months. Two other health facilities in the study area (one government facility and one faith based facility) were not included in the study because very few pregnant women attended ANC services there. On average, 12 randomly selected pregnant women were interviewed per visit (min-max = 1-21) adding up to a total of 43 pregnant women interviewed per health facility (min-max = 28-79).

### Data collection instruments

#### Health worker in-depth interviews

The interviews explored a) health workers' knowledge and attitudes towards the IPTp strategy, and b) their perception on factors influencing IPTp delivery. A semi-structured interview guide was designed in English and piloted outside the study area. The interview guide ensured the coverage of the main topics, but interviewees were encouraged to express their opinions and concerns freely. The interviews were translated and administered in Swahili. All but one of the interviews were tape-recorded after obtaining written informed consent from the health workers.

#### Exit interviews with ANC attendees

Participants were interviewed by two trained local female field workers after obtaining the women's informed consent. The main investigator supervised data collection during the two first rounds and checked the completeness of the data collected.

Information was collected using a structured questionnaire. The questionnaire was designed in English, translated to Swahili, back-translated and pre-tested outside the study area. Since the exit interviews were conducted in the frame of a larger research project to investigate pregnant women's access to ANC and IPTp services, questions were related to a) demographic characteristics, b) knowledge about ANC services and motivation to attend the ANC clinics, and c) ANC service utilization, but also inquired d) women's knowledge on malaria prevention, and e) their IPTp use. Additionally, data on the number and timing of ANC visits and IPTp doses received were copied from the ANC cards in order to avoid recall bias.

### Data analysis

The in-depth interviews with the health workers were transcribed and translated into English. Analysis was done using the qualitative data management software MaxQDA2 (Lucanus GmbH, Berlin, Germany). Text segments were coded into categories using qualitative content analysis [45]. Key themes emerging around health workers' knowledge on and experiences with the IPTp strategy were cross-tabulated in order to explore differences between and within health facilities.

From the exit interviews mainly ANC card data were analysed for the purpose of this study. Demographic data on marital status, socio-demographic status and level of education will not be investigated in this paper as several previous studies from Tanzania and elsewhere have not demonstrated any association between these characteristics and IPTp uptake [1,27,30,35].

Data were double-entered using Microsoft Access, validated with EpiInfo version 3.3.2 (EpiInfo Association, Denmark) and analysed in Stata 10 (StataCorop, College Station, Texas, USA). Descriptive results are presented using medians and interquartile ranges (IQR = 75^th^-25^th ^percentile). Differences in proportions were calculated using chi-square-test and Fisher's exact test where appropriate. For all statistical tests a two-sided P-value less than 0.05 was considered significant.

Firstly, women's timing of ANC visits and their eligibility for IPTp according to national guidelines were assessed. Women were assumed to be eligible for IPTp doses if they attended the ANC clinic between 20-24 weeks and/or 28-32 weeks of gestation. Secondly, health workers' IPTp delivery and its timing were evaluated in relation to the national guidelines by using ANC card records on women's gestational age at the time of IPTp receipt. In order to avoid biases resulting from the inclusion of women who according to guidelines were not yet eligible for IPTp due to their low gestational age, IPTp uptake was analysed for two sub-samples: 1) women of at least 20 weeks of gestation and 2) women of at least 28 weeks of gestation. Thirdly, it was estimated how the two different schedules of the national IPTp policies available in the malaria diagnosis and treatment guidelines and the FANC guidelines fit with women's timing of ANC attendance and health workers' IPTp delivery and timing. For simplicity, the two IPTp guidelines will be denoted as *restrictive *IPTp guidelines (recommending IPTp administration between 20-24 and 28-32 weeks of gestation) and *simplified *IPTp guidelines (recommending two doses of IPTp after quickening and at least one month apart) (Figure 1). For the *simplified *IPTp guideline, correct IPTp delivery was defined as two doses administered at any time between 20 weeks of gestation, which is the time when most women have already recognized first movements of the foetus [46], and 40 weeks of gestation as long as they are one month apart. Finally, the combined impact of women's timing of ANC attendance and health worker's delivery and timing of IPTp on the effectiveness of the IPTp strategy was assessed for each of the two national IPTp guidelines. Only women who had made at least two ANC visits were included (N = 189) in the sample. Effectiveness loss was calculated in steps by calculating: 1) the number of women who attended ANC services twice timely according to the guidelines in order to be eligible for IPTp doses; and 2) the number of women who attended timely and received one or two doses of IPTp.

### Ethical considerations

The study was carried out in the frame of the ACCESS Programme which has been cleared by the National Institution for Medical Research of Tanzania (NIMR/HQ/R.8c/Vol. I/66) [47]. The study was also approved by the review boards of the Swiss Tropical and Public Health Institute (STPH), formerly known as Swiss Tropical Institute (STI), and the Ifakara Health Institute (IHI), formerly known as Ifakara Health Research and Development Centre (IHRDC). The study was discussed and approved by the district coordinators for Reproductive-and-Child-Health (RCH) and staff in-charge was asked for permission to conduct the study at their facilities. Oral or written consent was obtained from all pregnant women and health-workers participating in the study after explaining the purpose of the study to them and informing them of their right to withdraw at any time. Health workers were asked for the permission to tape-record the interviews.

## Results

### Study population

A total of 18 health workers participated in in-depth interviews. They had between 0.5 and 24 years of working experience at the specific health facility and obtained different professional qualifications: 44% (8/18) were medical attendants (attended a one-year training), 22% (4/18) were MCH (Mother and Child Health) Aides (attended a two-year training), 22% (4/18) were certificated Nurse Midwifes (attended a four-year nursing training or upgrade training) and 6% (1/18) were Nursing Officers (attended a six-year training in nursing).

Of the 440 pregnant women who participated in the exit interviews, 10 women were excluded from the analysis because their ANC cards did not contain information on gestational age. Table 1 summarizes the characteristics of the 430 participants. The median age of the respondents was 25 years (IQR = 31-21) and 15% (62/430) of all women were 19 years old or younger. Among all women, 52% (225/430) had completed 7 years of primary school, 88% (377/430) were married and 20% (84/430) were in their first pregnancy. Overall, the median number of pregnancies was 3 (IQR = 5-2, including the current pregnancy). On the day of the interview, the majority of women were either between 20-24 weeks (43%, 185/430) or between 28-32 weeks of gestation (30%, 128/430). Median gestational age at the time of the interview was 24 weeks (IQR = 28-20). Among all participants, 56% (241/430) attended the ANC clinic for the first time in their current pregnancy on the day of the interview. The median number of ANC visits was one (IQR = 2-1).

**Table 1:** Socio-demographic characteristics of the respondents

Characteristic	%	(n) N = 430
Age groups		
<20	15	(62)
20-24	33	(132)
25-29	20	(83)
30-34	18	(72)
35-39	11	(44)
>39	3	(12)

Education level*		
No education	24	(103)
Incomplete primary	23	(99)
Primary +	52	(225)

Marital status		
Married or living with partner	88	(377)
Single or separated	12	(50)

Parity		
Para 1	19	(84)
Para 2-4	54	(231)
Para 5+	27	(115)

Gestational age at day of interview		
<20 weeks	14	(60)
20-24 weeks	43	(185)
25-27 weeks	3	(14)
28-32 weeks	30	(128)
>32 weeks	10	(43)

No. of women attending the first time	56	(241)
No. of women attending the second time	27	(117)
No. of women attending the third or more time	17	(72)

*does not add up to 100% due to missing values

### Knowledge about IPTp among health workers

Health workers' awareness about the IPTp strategy was high, but concentrated on the *restrictive *IPTp schedule. The majority of interviewees (11/18) explained in line with the *restrictive *IPTp guidelines, that IPTp should be administered between 20-24 weeks and 28-32 weeks of gestation, or in the fifth and seventh months. However, some health workers were confused as to when and how many doses of IPTp to administer. A third of the health workers (7/18) reported a deviant IPTp schedule: two health workers who were working in the same health facility indicated that they start giving the first SP dose after 16 weeks of gestation; another three health workers coming from two health facilities said they would give a total of three doses of SP, but were confused as to when to deliver them; three other health workers from different facilities reported giving SP up to the 34^th ^and 36^th ^week respectively. All seven health workers who reported deviant IPTp schedules stated that they had been instructed in a seminar to extend the schedule or increase the number of doses.

### Knowledge and attitudes about malaria prevention among pregnant women

Awareness about IPTp was not high among the pregnant women interviewed. 34% (148/430) of the pregnant women mentioned IPTp spontaneously as a service that they should receive at the ANC clinics. Asking the participants specifically about means of malaria prevention that they expect to get at the ANC clinic, SP/Fansidar/antimalarial was stated by 64% (277/430). 17% (75/430) did not know. Most women were aware that they get malaria prevention for their own (13%, 56/430) or for the child's protection (27%, 118/430) or for both mother and child (36%, 155/430). However, almost a quarter of the participants (23%, 99/430) answered the question "why do they provide you with malaria prevention when you go to the ANC clinic" with "I don't know".

### Pregnant women's timing of ANC visits

**Figure 2:**
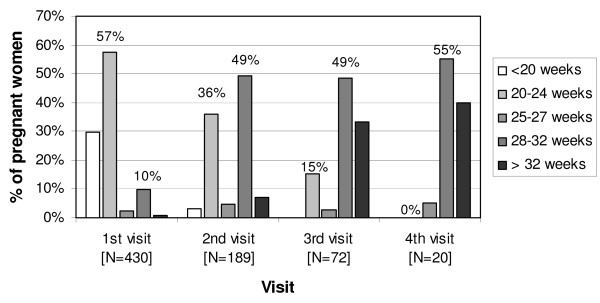
Women's attendance by gestational age and visit

Pregnant women's ANC attendance was in line with the *restrictive *IPTp schedule as most of them attended at 20-24 and 28-32 weeks of gestation. Figure 2 presents pregnant women's gestational age at their first, second, third and fourth ANC visit. It illustrates that the majority of women (57%, 247/430) were already between 20-24 gestational weeks at their first visit. Median gestational age at the first visit was 20 weeks which is consistent with the national average of 20.1 gestational weeks among health facility users [48]. Around half of the women were between 28-32 weeks of gestation at their second, third or fourth visit.

**Figure 3:**
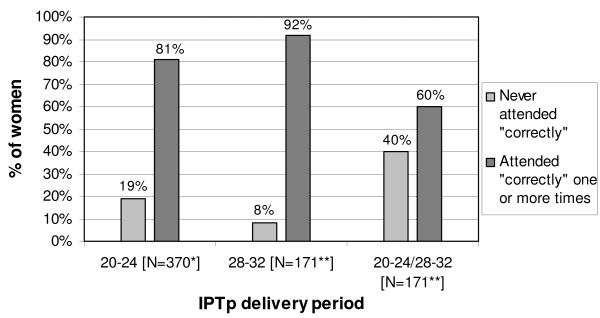
**Women's timely attendance according to the restrictive guidelines**. *No. of women of at least 20 weeks of gestation, **No. of women of at least 28 weeks of gestation

The majority of pregnant women attended at least once during a time period in which they were eligible for a first and/or a second IPTp dose at their ANC visits (Figure 3). The number of visits made during the two IPTp delivery periods specified by the *restrictive *guidelines was analysed for the two sub-samples of women of at least 20 and 28 weeks of gestation. 81% (299/370) of the women at 20 and more weeks of gestation attended the ANC clinic in a way that they were at least once eligible for a first dose of IPTp between 20-24 weeks. 15% (55/370) were not eligible for IPTp because they delayed ANC attendance until after 24 weeks of gestation. Among the women who were at least 28 weeks of gestation 92% (157/171) were eligible at one or more ANC visits for an IPTp dose between 28-32 weeks of gestation. The fact that only 8% of the women did not attend between the 28-32 weeks of gestation indicates that visits were more timely during the second delivery period compared to the first delivery period. Analysis for all women who had reached 28 weeks of gestation showed that 60% (102/171) attended both between 20-24 and 28-32 weeks of gestation and were therefore eligible for two doses of IPTp.

In summary, this analysis shows that women attended in accordance with the *restrictive *guidelines and ANC attendance initiated later than 24 weeks of gestation was low. Thus, the majority of women attended at the right time to receive IPTp during the two IPTp delivery periods.

### Health workers' IPTp delivery and its timing

ANC card records showed that delivery coverage was high for one IPTp dose but low for the complete course of two IPTp doses. Table 2 illustrates that among the women who were 20 weeks of gestation and older, 21% (79/370) had not yet received any IPTp dose and 79% (291/370) were given one or more doses of IPTp. However, only 27% (46/171) of the women who were at least 28 weeks of gestation had received two or more doses.

**Table 2:** IPTp delivery

Characteristic	%	(n/N)
Use of IPTp-SP		
None	21.4	(79/370)*
At least one	78.7	(291/370)*
At least two	26.9	(46/171)**

Gestational age at 1^st ^IPTp-SP dose		
<20 week	4.7	(14/299)
20-24 weeks	72.9	(218/299)
>24 weeks	22.4	(67/299)

Gestational age at 2^nd ^IPTp-SP dose		
<28 weeks	11.8	(6/51)
28-32 weeks	84.3	(43/51)
>32 weeks	3.9	(2/51)

*No. of women of at least 20 weeks of gestation, **No. of women of at least 28 weeks of gestation

Analysis of IPTp delivery by women's gestational age showed that the timing of the IPTp delivery was in accordance with the *restrictive *IPTp guidelines (Table 2): most women received IPTp during the specific periods of 20-24 and 28-32 weeks of gestation. 73% (218/299) of the women who had received a first dose of IPTp got it between 20-24 weeks; 5% (14/299) received it before and 22% (67/299) after this period. Among the women who had received a second dose of IPTp, 84% (43/51) got it between 28-32 weeks of gestation. Among the total SP doses administered to pregnant women only 11% (38/352) were distributed outside the two delivery periods.

However, although most women attended the ANC clinics during the required periods of 20-24 and/or 28-32 weeks of gestation, several women did not receive IPTp from the health workers, especially when attending between 28-32 weeks of gestation. Using data from the two subgroups, Table 3 reports the number of women who attended ANC on time to be eligible for a dose of IPTp and actually received IPTp. Among the women who were at least 20 weeks of gestation, 81% (299/370) attended between 20-24 weeks. Out of those, 73% (218/299) received IPTp when they attended between 20-24 weeks. Among the women who were at least 28 weeks of gestation, 92% (157/171) of the women attended the ANC clinics at least once between 28-32 weeks. However, only 55% (87/157) of them got an IPTp dose either as a first or a second dose. This suggests that health workers deliver IPTp significantly less well between 28-32 week of gestation than between 20-24 weeks (55.4% vs. 72.9%; p < 0.001). Consequently, the coverage for two doses of IPTp was low: among those who attended during both IPT delivery periods (at 20-24 weeks and 28-32 weeks of gestation) only 30% (30/102) actually received two doses of IPTp.

**Table 3:** Women's timing of ANC attendance and SP delivery

	No. of women attending between 20-24 weeks and/or 28-32 weeks of gestation at any visit		No. of women receiving SP between 20-24 weeks and/or 28-32 weeks of gestation at any visit	
	
	% (CI 95%)	n/N	% (CI 95%)	n/N
20-24 weeks of gestation	80.8 (76.4-84.7)	299/370*	72.9 (67.5-77.9)	218/299

28-32 weeks of gestation	91.8 (86.6-95.5)	157/171**	55.4 (47.3-63.3)	87/157

20-24 AND 28-32 weeks of gestation	59.7 (51.9-67.1)	102/171**	29.4 (14.7-49.4)	30/102

*No. of women of at least 20 weeks of gestation, **No. of women of at least 28 weeks of gestation

To summarize, analysis of health workers' IPTp delivery showed that they adhered well to the *restrictive *guidelines as far as timing of IPTp delivery is concerned. However, women attending ANC clinics between 28-32 weeks of gestation were 24% (100%-55.4%/72.9%) less likely to receive a dose of IPTp than women attending between 20-24 weeks of gestation. Consequently, second dose coverage was low.

### IPTp guidelines compared

Opportunities to reach high IPTp coverage levels were not only missed because of undelivered IPTp doses at the health facilities, but also due to the poor implementation of the IPTp strategy. In fact, these two aspects may be closely interlinked. Although WHO's simplified IPTp schedule from 2004 has been integrated into the Tanzanian FANC guidelines the same year, health workers at the ANC clinics still followed the former IPTp policy and administered IPTp during two restrictive periods in 2007 and 2008. Using the information on number and timing of ANC visits and received IPTp doses among the study population, an estimate of the increase in IPTp coverage that could potentially have been gained by implementing the *simplified *WHO guidelines was calculated. Table 4 summarizes the additional number of women from the survey sample who would have been eligible to IPTp according to the *simplified *IPTp guidelines recommended by the WHO compared to the *restrictive *IPTp schedule currently practiced at the ANC clinics. Women's first visit was analysed to assess the additional number of women eligible for one IPTp dose and women's first two, three and four visits were examined to assess the number of additional women eligible for two doses of IPTp. Among the women who attended the ANC clinic for the first time, a significantly higher proportion of women would have been able to receive one IPTp dose (69% vs. 57%, p < 0.001). At the first two visits, IPTp coverage could have been increased by 19 percentage points (p < 0.001); and at the first three visits by 20 percentage points (p < 0.001) by adhering to the *simplified *guidelines. At the level of four ANC visits no difference between the two guidelines was observable due to the small sample size (N = 20).

**Table 4:** Number of women eligible for IPTp according to the two guidelines

	Restrictive guidelines (20-24, 28-32 weeks of gestation)		Simplified guidelines (20-40 weeks of gestation, one month apart)		
		
	% (95% CI)	n/N	% (95% CI)	n/N	p-value*
Visits	No. of women eligible for one dose of IPTp				
First visit	57.4 (52.6-62.2)	247/430	69.5 (64.9-73.8)	299/430	<0.001

	No. of women eligible for at least two doses of IPTp				
First two visits	40.2 (33.2-47.6)	76/189	59.3 (51.9-66.3)	112/189	<0.001
First three visits	70.8 (58.9-80.9)	51/72	90.3 (81-96)	65/72	<0.001
First four visits	90.0 (68.3-98.7)	18/20	95.0 (75.1-99.9)	19/20	1.0

* P-values based on chi-square test and Fishers exact test at four visits

Furthermore, according to the *simplified *guidelines health workers would not only have been able to deliver two IPTp doses at the right time to more women, but could also have administered more than two IPTp doses to 20% (37/189) of the women who attended the ANC clinic at least twice.

In summary, through the implementation of the *simplified *guidelines recommended by WHO IPTp coverage at their first, second and third ANC visits could have been increased by 12 to 20 percentage points. Moreover, the number of delivered doses of IPTp could be increased.

### Lost effectiveness of IPTp strategy

**Figure 4:**
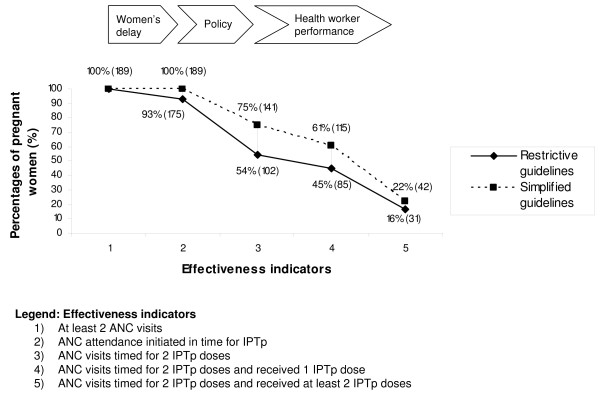
Effectiveness loss of the IPTp strategy due to individual, facility and policy factors

Women's timing of ANC attendance, health workers' IPTp delivery and policy issues all influence IPTp coverage levels. Based on the collected ANC record data, Figure 4 illustrates the impact of these bottlenecks on the effectiveness of the IPTp strategy for the two available IPTp guidelines (*restrictive *vs. *simplified*): Out of 189 women who made at least two ANC visits, 7% started ANC attendance too late to receive the first IPTp dose timely according to the *restrictive *guidelines. According to the *simplified *guidelines all of them would have received IPTp in time. 54% (102/189) of the women who made two or more ANC visits, would have been eligible for two IPTp doses according to the *restrictive *guidelines while 75% (141/189) of them attended timely according to the *simplified *guidelines (p < 0.001). Given the current IPTp delivery practices observed among health workers, 45% (85/189) of the women were given at least one dose of IPTp and 16% (31/189) were given two doses of IPTp at the correct time according to the *restrictive *guidelines. Applying the *simplified *guidelines, 61% (115/189) received at least one dose and 22% (42/189) at least two doses of IPTp at the right time. This indicates that effectiveness of the IPTp strategy is lost especially through policy issues and health worker practices.

## Discussion

The study illustrated the combined effect of women's timing of ANC attendance, health worker's IPTp delivery and different delivery schedules of national IPTp guidelines on IPTp coverage. This is in line with findings of previous studies from Tanzania showing that low IPTp coverage levels can not be attributed solely to women's late enrolment to ANC [26,30]. Instead, health worker's IPTp delivery practices and unclear IPTp guidelines led to lost effectiveness of the IPTp strategy. Thus, solutions need to be found at individual, facility and policy level if the government of Tanzania aims at reaching at least 80% of pregnant women with two IPTp doses [22].

Compared to the *restricted *IPTp guidelines, pregnant women initiated ANC attendance late, but still in time to receive a first IPTp dose. Pregnant women in Tanzania are recommended to attend ANC clinics for the first time at 16 weeks of gestation [23]. Most women started ANC attendance in their second trimester around 20 weeks of gestation. This is consistent with the national average among facility users [48] and with findings from other studies [26,28,30,31,35]. However, contrary to health workers' perception and assumptions in the literature [29,33,49,50], women's late ANC enrolment did not interfere with the IPTp schedule. This has also been stressed by other studies [26,30,31]. Only 15% of the women started ANC attendance after 24 weeks of gestation and were therefore according to the *restrictive *guidelines no longer eligible for a first dose of IPTp. Overall, women's timing of ANC visits matched well with the *restrictive *IPTp schedule that was practiced in health facilities. The majority of the participants attended the ANC clinic at least once between 20-24 weeks and between 28-32 weeks of gestation. This shows that women's ANC attendance follows health workers' instructions and was not the main cause for low IPTp levels. IPTp coverage could theoretically have been high. The high proportion of women attending during the specific periods is not surprising as women are given return dates by the health workers. Although women's knowledge about the timing of IPTp was not investigated, it can be presumed that women rely on health workers to correctly administer drugs [18,49]. Findings of Marchant et al. [30] support this assumption: over 90% of the women who had not received a dose of IPTp said that health workers had not offered it to them. The participants' knowledge on malaria prevention and its effects was not very high. Almost a quarter of the women did not know why they were supposed to get malaria prevention at the ANC clinic. Pregnant women's knowledge concerning IPTp but also women's power and ability to actively demand IPTp and to protect themselves from erratic timing or missed delivery of IPTp [36] needs to be improved.

Health workers' IPTp delivery was characterized by its focus on the *restrictive *IPTp guidelines and by low delivery levels between 28-32 weeks of gestation. Timing of IPTp delivery was in accordance with the *restrictive *IPTp schedule as most IPTp doses were delivered within the specified periods of 20-24 weeks and 28-32 weeks of gestation. Only 11% of the total IPTp doses were administered outside these periods. Awareness of the IPTp strategy was high among the health workers, however, the concurrent existence of different IPTp guidelines led to some confusion about when and how many IPTp doses to administer.

Consistent with findings from several other studies [26,27,29,31,48], most women in the study got a first dose of IPTp, but many did not receive a complete course of two IPTp doses. HMIS data collected in the study area in 2008 provided a similar picture: less than half of the women who were given a first IPTp dose, also received a second one. Health workers' low performance provides one possible explanation [50,51]: Observations of ANC consultations in the context of a study on quality of care showed that return visits were usually of very short duration and reduced to the most basic examinations such as abdominal palpation and the measurement of blood pressure and weight (Gross et al., submitted to BMC Pregnancy and Childbirth). In this context, IPTp as well as the delivery of other drugs and lab examinations might easily be skipped or forgotten. This pattern has also been reported in a study from Malawi [33]. Given the shortages of SP often observed in Tanzania [26,30,52] health workers' rationing of SP, especially of the second IPTp dose, might represent another possible explanation. Information on SP stock-outs was not collected at the time of the study as it would only have served to explain the missed delivery of the most recent IPTp doses. Monitoring SP stocks at the health facilities through the collection of end of month drug stock data was not possible due to low quality of record keeping by the facility staff. However, a quality of care study conducted in October 2008 in the same area showed that all the selected health facility except one had SP available in the three preceding months (unpublished data, ACCESS Programme). As this study leaves open questions on why health workers delivered the second IPTp dose significantly less well than the first one, it calls for future studies on health workers IPTp delivery practices.

IPTp delivery practices at ANC clinics in the study area and other regions of Tanzania differ critically from the *simplified *WHO recommendations to distribute SP to all pregnant women twice after quickening and one month apart [26,30]. The government's failure to implement the *simplified *IPTp schedule caused on the one hand confusion among health workers due to the concurrent existence of different and contradictory IPTp policies. On the other hand it represents a missed opportunity for high coverage levels of this important intervention. Calculations based on the *simplified *guidelines suggest that IPTp coverage could potentially be increased by up to 20 percentage points if IPTp delivery were no longer limited to the narrow gestational range of 20-24 and 28-32 weeks. Better outcomes are also to be expected as health workers' difficulties to assess gestational age is taken into account [19].

The Ministry of Health should therefore overcome inconsistent IPTp messages and advocate one clear IPTp recommendation. The study showed that implementing the *simplified *IPTp policy recommended by WHO has the potential to reach more pregnant women with the important intervention of IPTp. The fact that the *simplified *IPTp guidelines are already integrated in the Focused Antenatal Care guidelines highlights the need for training health workers on the new policy and disseminating the information to the periphery.

Not only coverage but also the number of administered doses could be easily increased through the implementation of the *simplified *guidelines. WHO currently recommends a three dose regimen for areas in which antenatal HIV prevalence exceeds 10% and where HIV-testing is not available [53]. Trials from Kenya, Malawi and Zambia showed that receiving at least three courses of IPTp was associated with a better protective outcome among HIV-positive pregnant women [15,54-56]. Tanzania so far continues with a two-dose regimen due to its relatively low HIV prevalence rates. However, if levels of parasite resistance continue to increase, alternative drugs for IPTp need to be urgently found [57]. Any replacement drug to SP will most likely require a more complicated drug regimen. Thus, achieving high coverage levels will become even more challenging.

Collecting data through exit interviews at health facilities imposed two main limitations on the study: Firstly, information on women's ANC attendance behaviour was incomplete. Most women were at the beginning or at the middle of their pregnancy and data were usually available for less than four ANC visits. Moreover, although data collection took place over several months and at different ANC clinics it might not be representative for other places and periods of the year. Secondly, the conduct of exit interviews may have resulted in improved health worker performance. Including ANC card data on services received at earlier visits certainly lessened this type of bias. In reality, having a record of IPTp receipt is neither a guarantee that the drug was given nor taken - despite the fact that IPTp should be provided under direct observation. However, comparing ANC records with women's self-report revealed no major inconsistencies.

Finally, between the interviews with the health workers and the exit interviews with ANC attendees was a time gap of one year, since the exit interview survey was conducted in order to verify and validate certain results from the health worker interviews. Although health workers did not receive any training on IPTp delivery in the meantime, the time order of the studies did not allow to investigate health workers' reasons for the lower level of second dose IPTp delivery.

## Conclusions

This study showed that effective IPTp delivery to pregnant women is hampered by the combined effect of women's timing of ANC attendance, health worker's IPTp delivery and different delivery schedules of national IPTp guidelines. In particular, the implementation of *simplified *IPTp guidelines will be critical for reaching the 80% target. Training on *simplified *IPTp messages should be reinforced as part of the extended FANC training of health workers to change IPTp delivery practices and increase coverage levels. Additionally, campaigns that provide educational messages on the risk of malaria during pregnancy and the usefulness of IPTp and that help to raise patients' awareness for their rights are required to increase pregnant women's power to demand IPTp and other ANC services and decrease their dependence on health workers. Lessons need to be learned on how to implement guidelines changes effectively and determinedly in order to be prepared for the implementation of a new drug for IPTp when drug resistance against SP has reached levels where IPTp with SP is no longer efficacious.

## Competing interests

The authors declare that they have no competing interests.

## Authors' contributions

KG was responsible for the design and implementation of the study, carried out the data collection, the data management and analysis, and wrote the manuscript. SA assisted with data management, statistical analysis and contributed to the interpretation and discussion of the findings in the manuscript. IM participated in the study design and data collection process and commented on the manuscript. BO, FK and JS supported the design and coordination of the study and contributed to the discussion of the manuscript. All authors have read and approved the final manuscript.
